# Supplementary material for: Silencing of the lncRNA Zeb2-NAT facilitates reprogramming of aged fibroblasts and safeguards stem cell pluripotency
Source: Nat Commun. 2018 Jan 8;9:94. doi: 10.1038/s41467-017-01921-6 (PMC5758807; doi:10.1038/s41467-017-01921-6)
Supplement: Supplementary file 1 — Supplementary Information [file 41467_2017_1921_MOESM1_ESM.pdf]

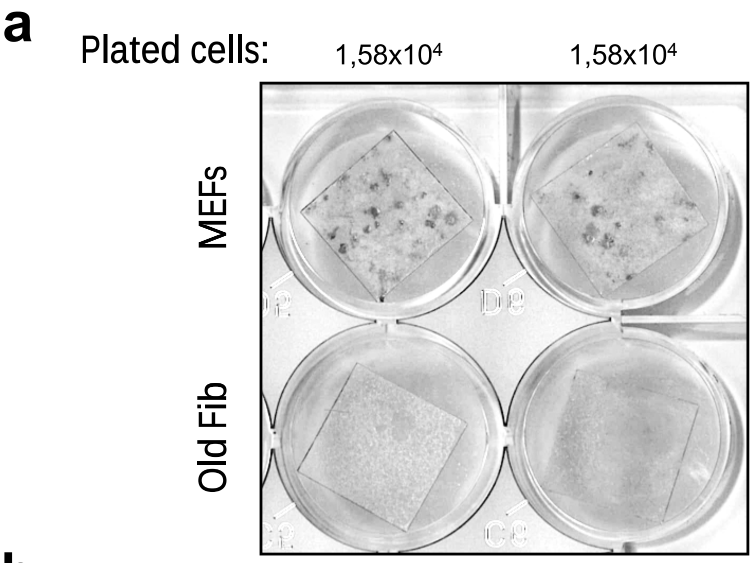

b

Quantification of Reprogramming Efficiency			
Experiment	Cells Plated	Colonies	Efficiency (%)
MEFs	15800	14 ± 2	0.09
Old Fib	15800	0	0

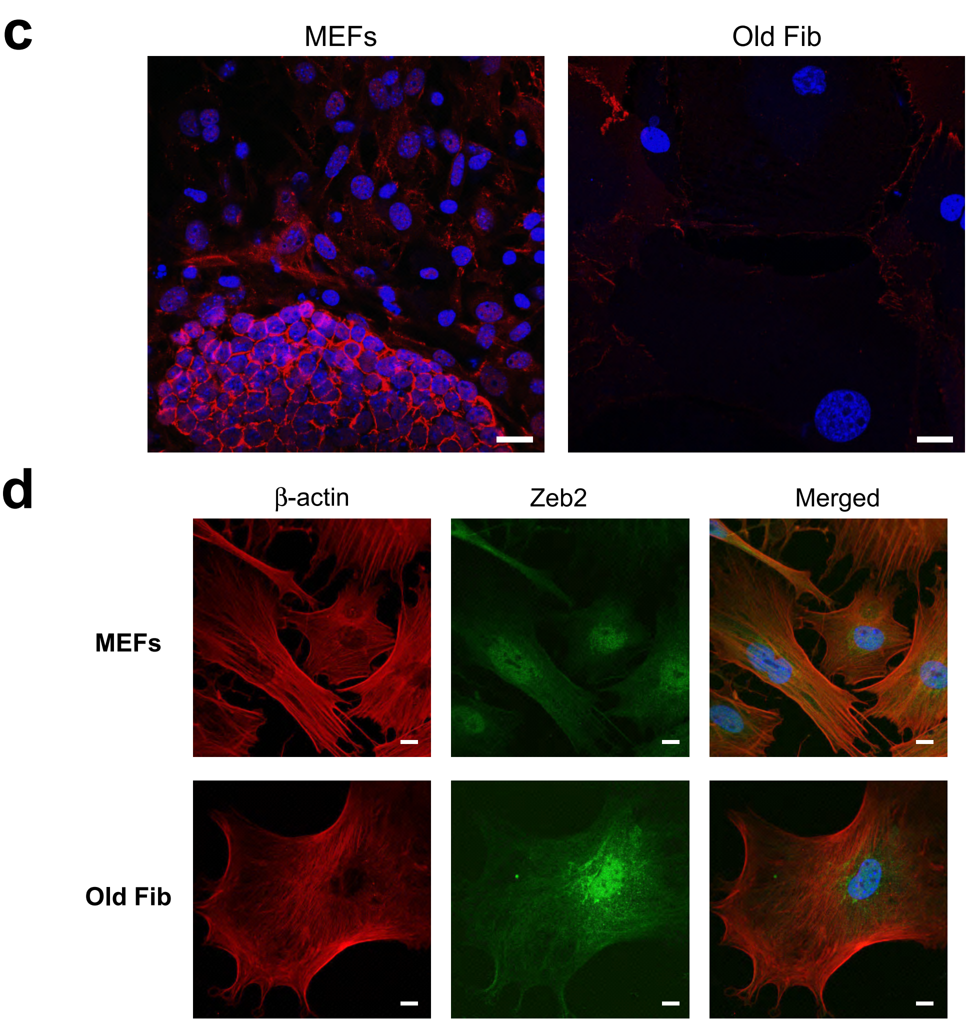

Supplementary Figure 1. Inefficient reprograming of old fibroblasts. **a,** The same number of early passage (P1) mouse embryonic fibroblasts (MEFs) and fibroblasts isolated from old (70-100 week-old) i4F mice were cultured in multiwell plates (1.58 x 10^4 cells per well) and induced with doxycycline. After 2 weeks of reprogramming, colonies were stained for alkaline phosphatase activity and counted. b, quantification of AP+ colonies. **c**, Immunofluorescence for β–catenin at day 12 after induction with doxycycline. A combined view with DAPI (4,6-diamidino-2-phenylindole) staining is shown (scale bars, 20 mm). **d,** Immunofluorescence for b-actin and Zeb2 in P1 cultures of MEFs and old fibroblasts. A combined view with DAPI is shown (scale bars, 10 mm).

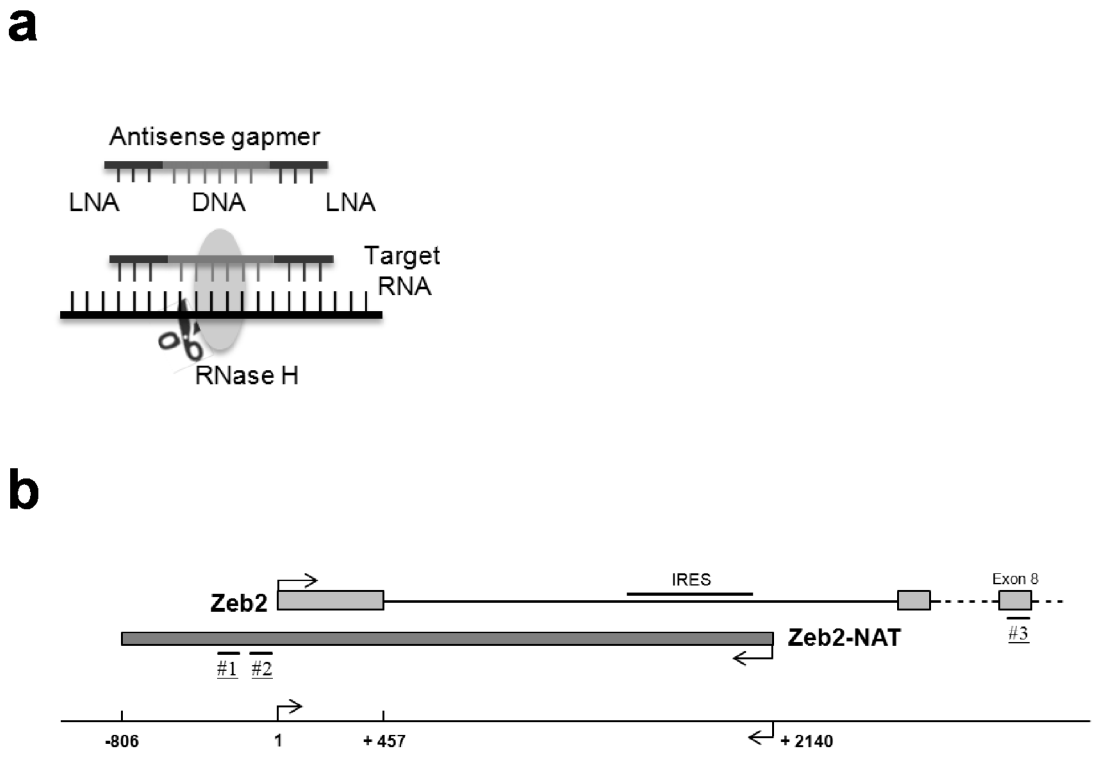

#1 5' – ACTCTGCAGGATTTA - 3'
#2 5' – TTAGTGATGAGGATA - 3'
#3 5' – GTTAGCCTGAGAGGAG - 3'

Supplementary Figure 2. Antisense oligonucleotides targeting Zeb2 and Zeb2-NAT. **a**, Schematics of the gapmers used in this study. **b**, The sequences and hybridization sites of LNA gapmers targeting Zeb2-NAT (#1, 2) and Zeb2 (#3) are indicated.

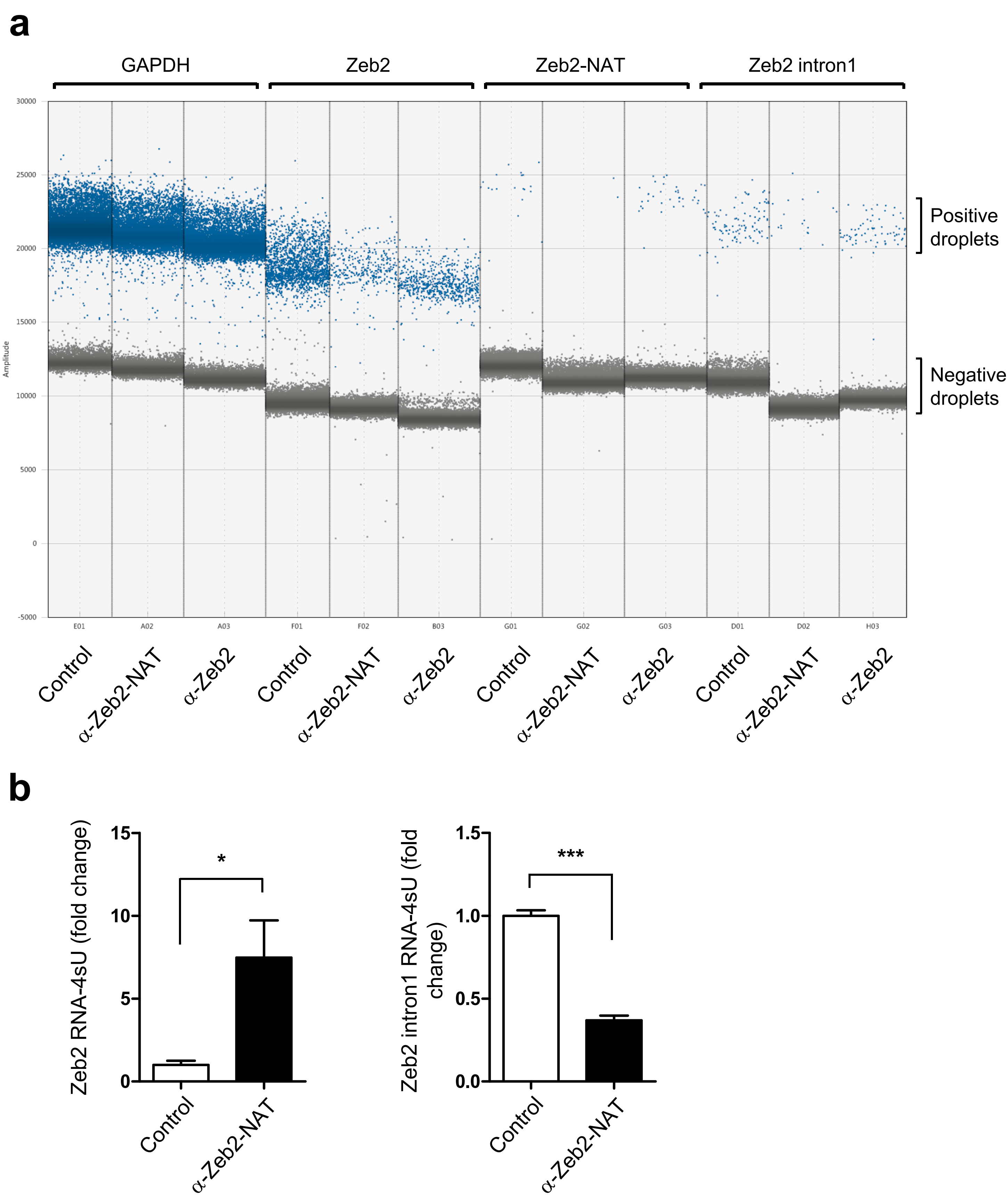

Supplementary Figure 3. Knocking down Zeb2-NAT affects Zeb2 splicing and transcription. Fibroblasts from old mice were transfected with either control LNA gapmers or oligonucleotides targeting Zeb2-NAT and Zeb2. **a,** droplet-digital-PCR analysis. **b**, After a labeling time of 30 minutes, newly-transcribed RNA-4sU was analyzed by qRT-PCR using primers Zeb2 #1 and Zeb2 intron for detection of total Zeb2 transcripts and transcripts with retention in the first intron, respectively. Student´s t-test (two-tailed) statistics, * p< 0,05; *** p<0,001; error bars represent standard deviation; 3 independent experiments were carried per condition.

a

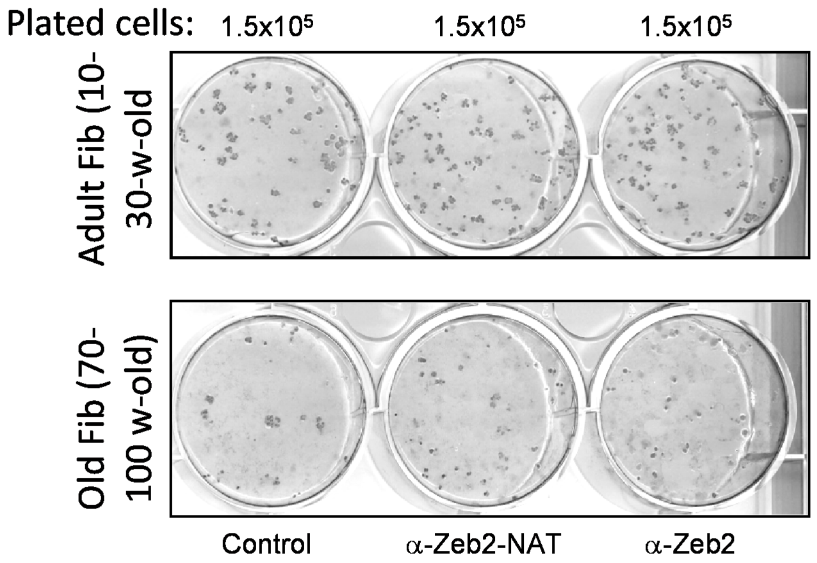

b

Quantification of Reprogramming Efficiency			
Experiment	Cells Plated	Colonies	Efficiency (%)
Adult Fib	150000	37 ± 5	0.025
Adult Fib + α-Zeb2-NAT	150000	59 ± 2	0.04
Adult Fib + α-Zeb2	150000	48 ± 2	0.03
Old Fib	150000	5 ± 5	0.003
Old Fib + α-Zeb2-NAT	150000	17 ± 11	0.01
Old Fib + α-Zeb2	150000	15 ± 10	0.01

c

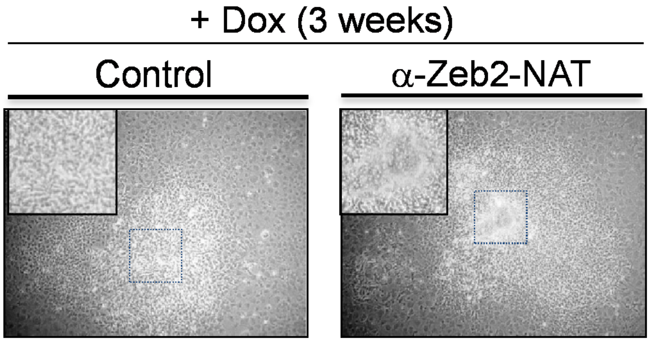

d

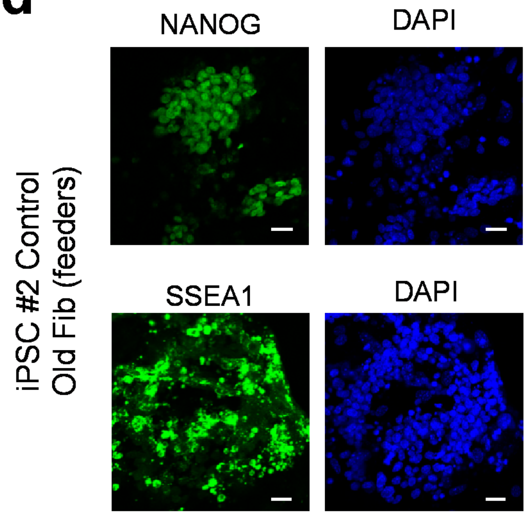

Supplementary Figure 4. Knocking down Zeb2-NAT enhances reprogramming of old fibroblasts. Fibroblasts from adult and old mice were transfected with either control LNA gapmers or oligonucleotides targeting Zeb2-NAT and Zeb2, and 24 hours later doxycycline was added to the culture to induce reprogramming. **a,** Representative culture plates incubated in the presence of doxycycline for 3 weeks and stained for Alkaline Phosphatase. **b,** quantification of AP^+ colonies. **c**, Morphology of reprogramming fibroblasts from old mice transfected with the indicated oligonucleotides after 3 weeks of doxycycline induction before culture in feeder-free medium. **d**, Immunofluorescence for Nanog and SSEA1 in representative colonies reprogrammed from untreated old fibroblasts initially cultured in the presence of feeder cells and passed 5 times in feeder-free medium before immunofluorescence; the corresponding images stained with DAPI are shown (scale bars, 20 mm).

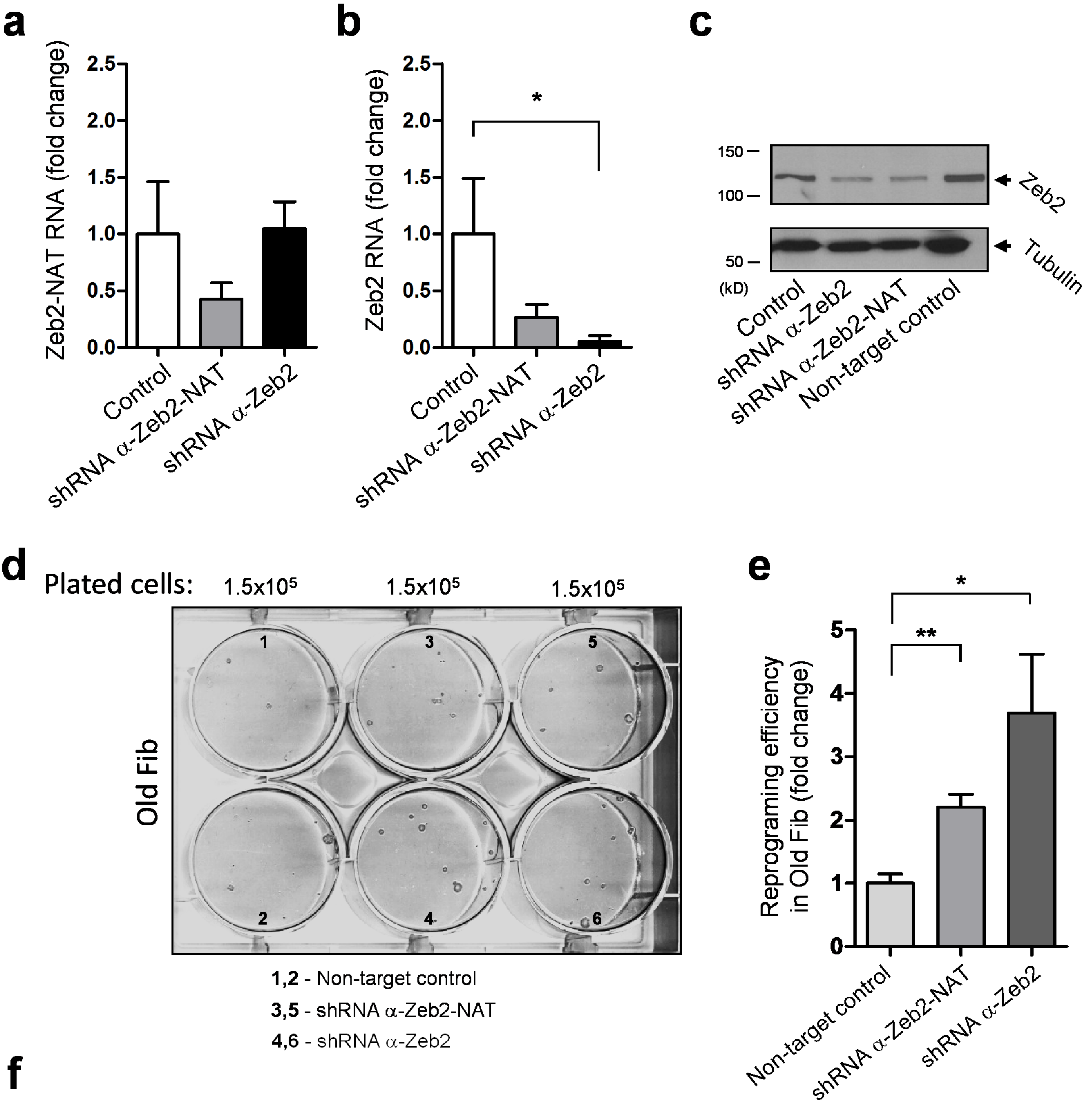

f

**Quantification of Reprogramming Efficiency**			
Experiment	Cells Plated	Colonies	Efficiency (%)
Old Fib + non-target control	150000	2 ± 1	0.0013
Old Fib + shRNA α-Zeb2-NAT	150000	5 ± 1	0.003
Old Fib + shRNA α-Zeb2	150000	8 ± 1	0.005

Supplementary Figure 5. Knockdown of Zeb2 and Zeb2-NAT expression using shRNAs. Fibroblasts from old mice were either non treated or infected with lentival particles containing a DNA sequence that codes for a shRNA targeting Zeb2-NAT, a shRNA targeting Zeb2, and a non-targeted control shRNA (sequences in Supplementary Table 1). **a,b,** qRT-PCR analysis of Zeb2-NAT and total Zeb2 RNA (primer Zeb2 #1, see Supplementary Table 2). Transcript levels were normalized to GAPDH mRNA and depicted as fold change relative the control condition. **c,** Immunoblot for Zeb2 and tubulin in total cell lysates. **d,** Representative reprogramming experiment stained for Alkaline Phosphatase after 3 weeks with doxycycline. **e,** Reprogramming efficiency estimated from the number of AP^+ colonies observed after 3 weeks in culture in the presence of doxycycline. **f,** Quantification of AP^+ colonies. For all graphics depicted, Student´s t-test (two-tailed) statistics, * $p< 0,05$, ** $p<0,01$; error bars represent standard deviation; at least 3 independent experiments were carried per condition.

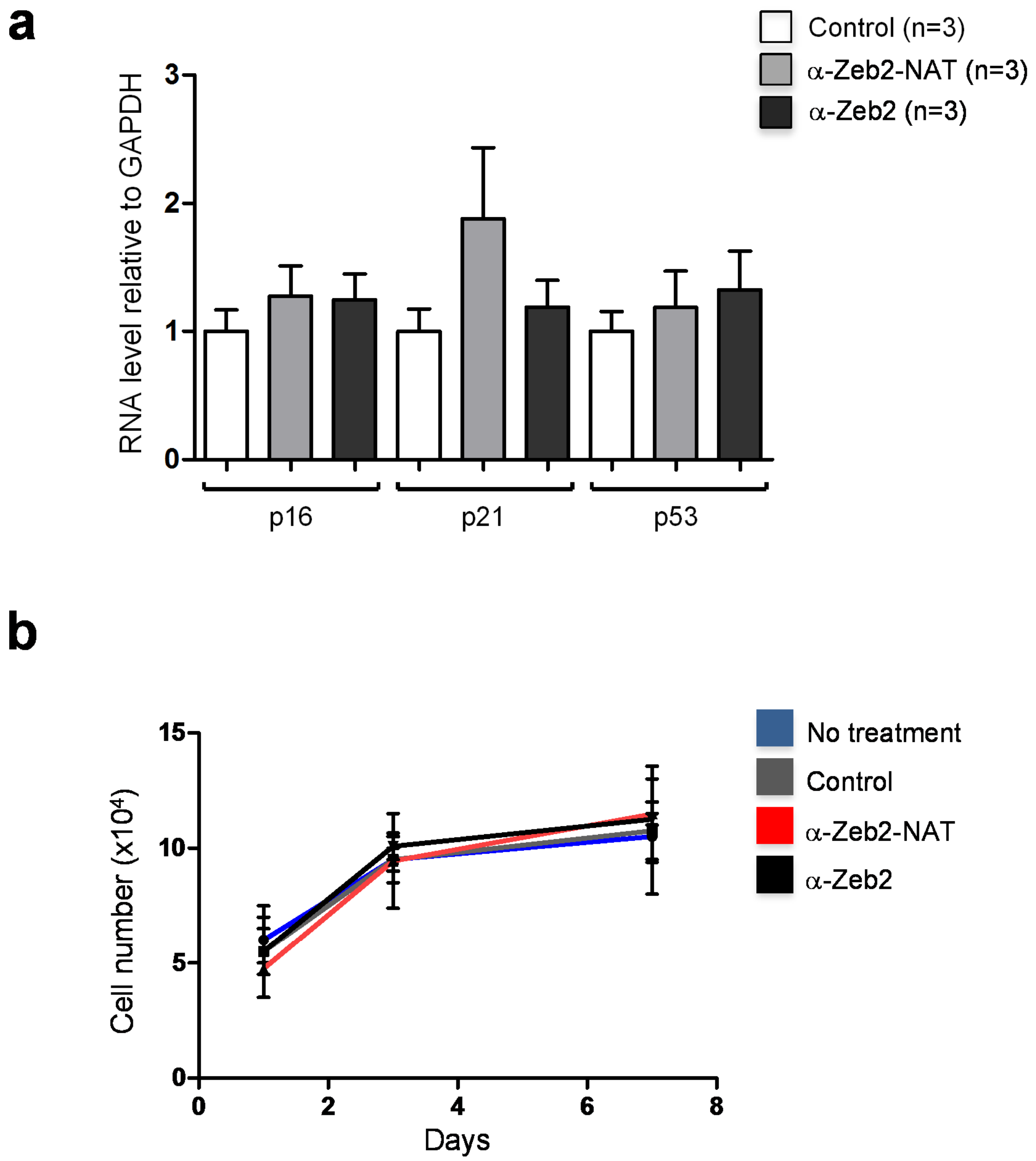

Supplementary Figure 6. Knockdown of Zeb2 and Zeb2-NAT does not affect cell proliferation. Fibroblasts from old mice were transfected with the indicated oligonucleotides. **a,** qRT-PCR analysis of p16, p21 and p53 mRNA. **b,** Cell number at the indicated days after transfection.

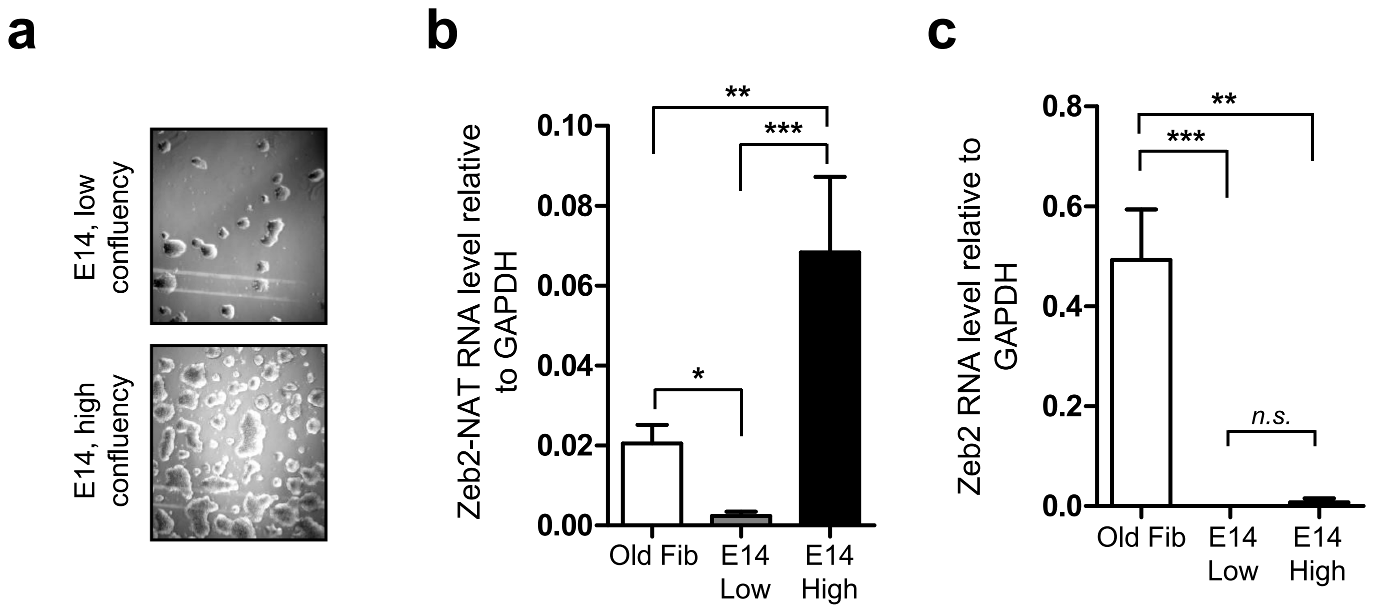

Supplementary Figure 7. Zeb2-NAT expression in ES cells changes rapidly in response to differentiation stimuli. a, Representative images of E14 cells grown at low or high confluency. **b, c,** qRT-PCR analysis of Zeb2-NAT and total Zeb2 RNA in old fibroblasts and E14 cells grown at low or high confluency. Student´s t-test (two-tailed) statistics, * p< 0,05, ** p< 0,01, *** p< 0,001; error bars represent standard deviation; at least 3 independent experiments were carried per condition.

a

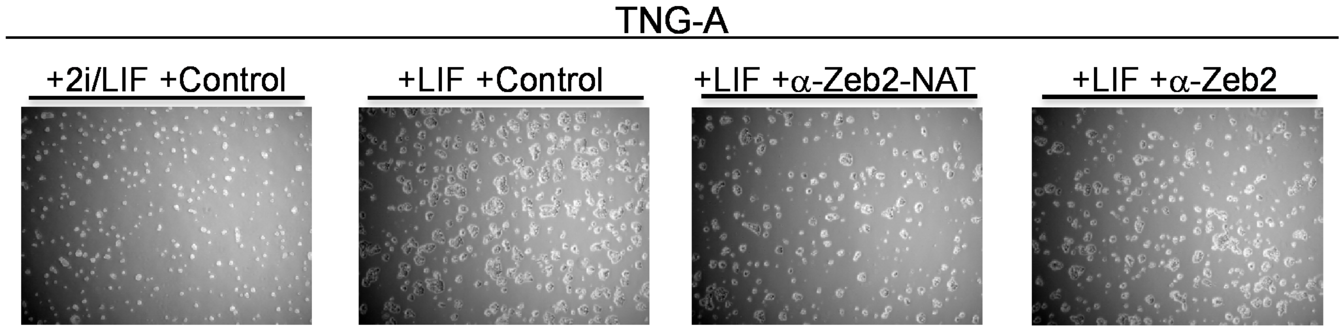

b

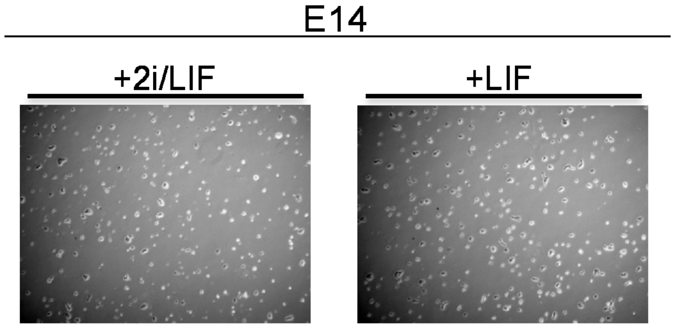

c

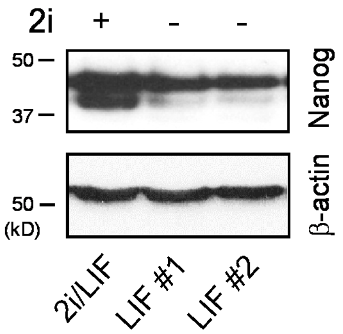

d

	Tumor Volume	ECTODERM					ENDODERM			MESODERM			
		skin and adnexal glands			*neural tissue*		*respiratory-type*		*GI tract*				
Group	mm^3	squamous epithelium	glandular epithelium (sebaceous/	melanocytes/ pigmented cells	primitive neuroepitheliu m	differentiate d nervous tissue	ciliated epithelium	goblet cells	esophagus to colon	bone	cartilage	muscle	adipose
E14+LIF	122,5	present	present	absent	absent	present	present	present	absent	absent	absent	present	no
E14+LIF/2i	224	present	present	absent	present	present	present	present	absent	present	present	present	no

Supplementary Figure 8. Removing 2i from the culture medium challenges ES cells. a, Representative images of TNG-A cells grown in the presence or absence of 2i for 48h; cells were previously transfected with either control LNA gapmers or oligonucleotides targeting Zeb2-NAT and Zeb2. **b,** Representative images of E14 cells grown in the presence or absence of 2i for 48h. **c,** Immunoblot for Nanog and β-actin in total cell lysates from E14 cells grown in the presence or absence of 2i. **d,** Characteristics of teratomas observed at 7 weeks after subcutaneous injection of $2x10^6$ E14 cells previously grown in the presence or absence of 2i.

a

Group	Animal ID	ECTODERM: *skin and adnexal glands*: squamous epithelium	ECTODERM: *skin and adnexal glands*: glandular epithelium (sebaceous/ apocrine)	ECTODERM: *neural tissue*: primitive neuroepitheliu m	ECTODERM: *neural tissue*: differentiate d nervous tissue	ENDODERM: *respiratory-type*: ciliated epithelium	ENDODERM: *respiratory-type*: goblet cells	MESODERM: bone	MESODERM: cartilage	MESODERM: muscle	MESODERM: adipose
TNGA LIF/2i	1	present	present	present	absent	present	present	present	present	present	absent
	2	absent	absent	absent	absent	absent	present	present	absent	absent	absent
TNGA LIF	1	absent	absent	absent	absent	absent	absent	absent	absent	absent	absent
	2	absent	absent	absent	absent	absent	absent	absent	absent	absent	absent
TNGA LIF α-NAT	1	present	present	present	present	present	present	present	present	present	present
	2	present	present	absent	present	absent	present	absent	absent	present	present
TNGA LIF α-Zeb2	1	present	present	absent	absent	absent	present	absent	present	absent	present
	2	absent	absent	absent	absent	present	absent	present	absent	absent	absent

b

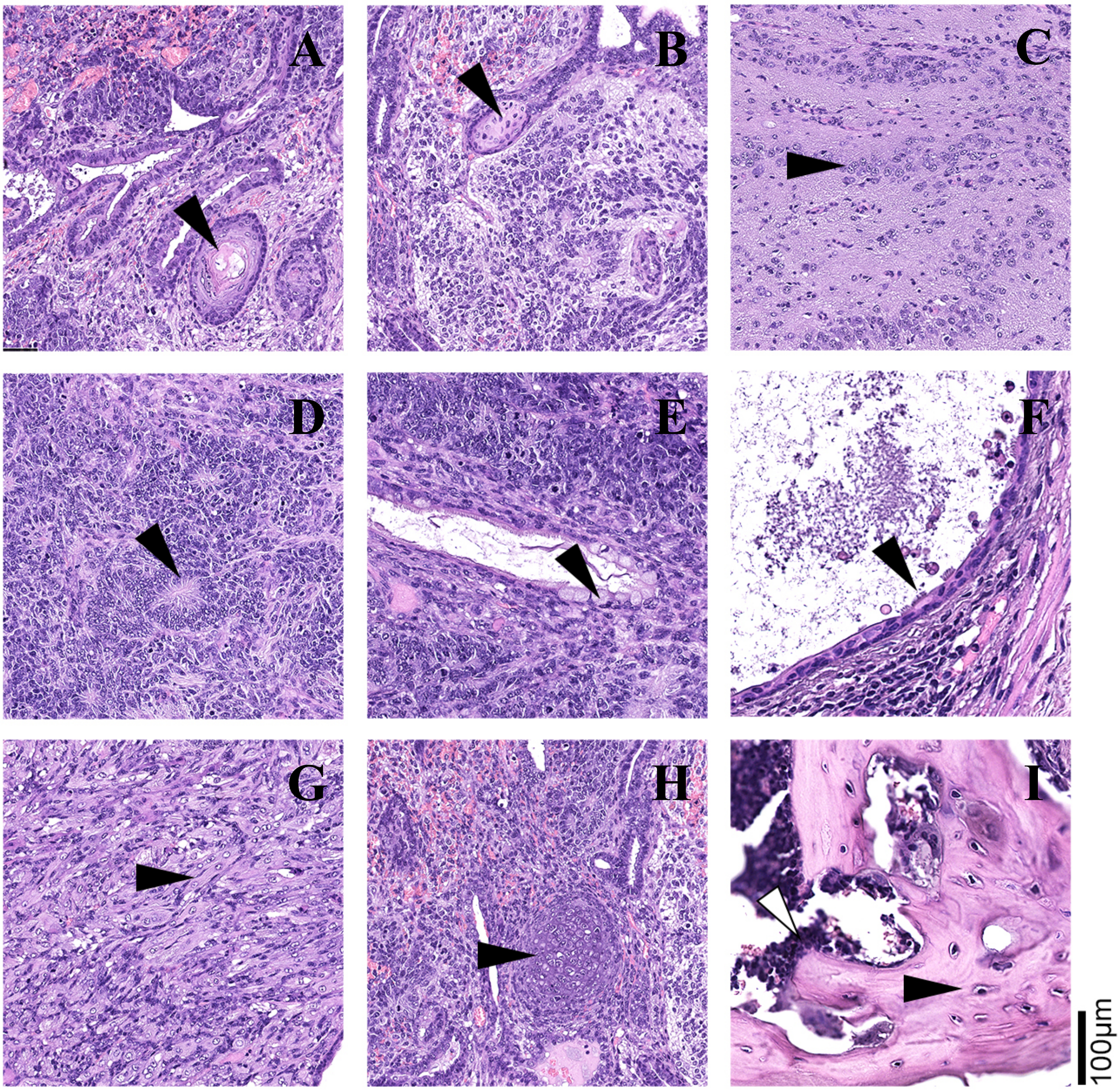

Supplementary Figure 9. Blocking Zeb2-NAT expression in TNG-A cells affects teratoma formation. TNG-A cells were either nontransfected or transfected with oligonucleotides targeting Zeb2 and Zeb2NAT, and then grown in the presence or absence of 2i. **a,** Characteristics of teratomas observed at 7 weeks after subcutaneous injection of 2x10^6 cells. **b,** Histological sections of teratomas stained with hematoxylin and eosin. Ectodermal components corresponding to skin and adnexa, including squamous (black arrow, A) and sebaceous epithelium (black arrow, B); differentiated nervous cells (black arrow, C) and primitive neuroepithelium, arranged in rosettes (black arrow, D). Endodermal components corresponding to respiratory-type epithelium, including ciliated (black arrow, E), and mucin-producing goblet cells (black arrow, F). Mesodermal components corresponding to muscle (black arrow, G), cartilage (black arrow, H) and bone and hematopoietic tissue (black and white arrow, respectively, I).

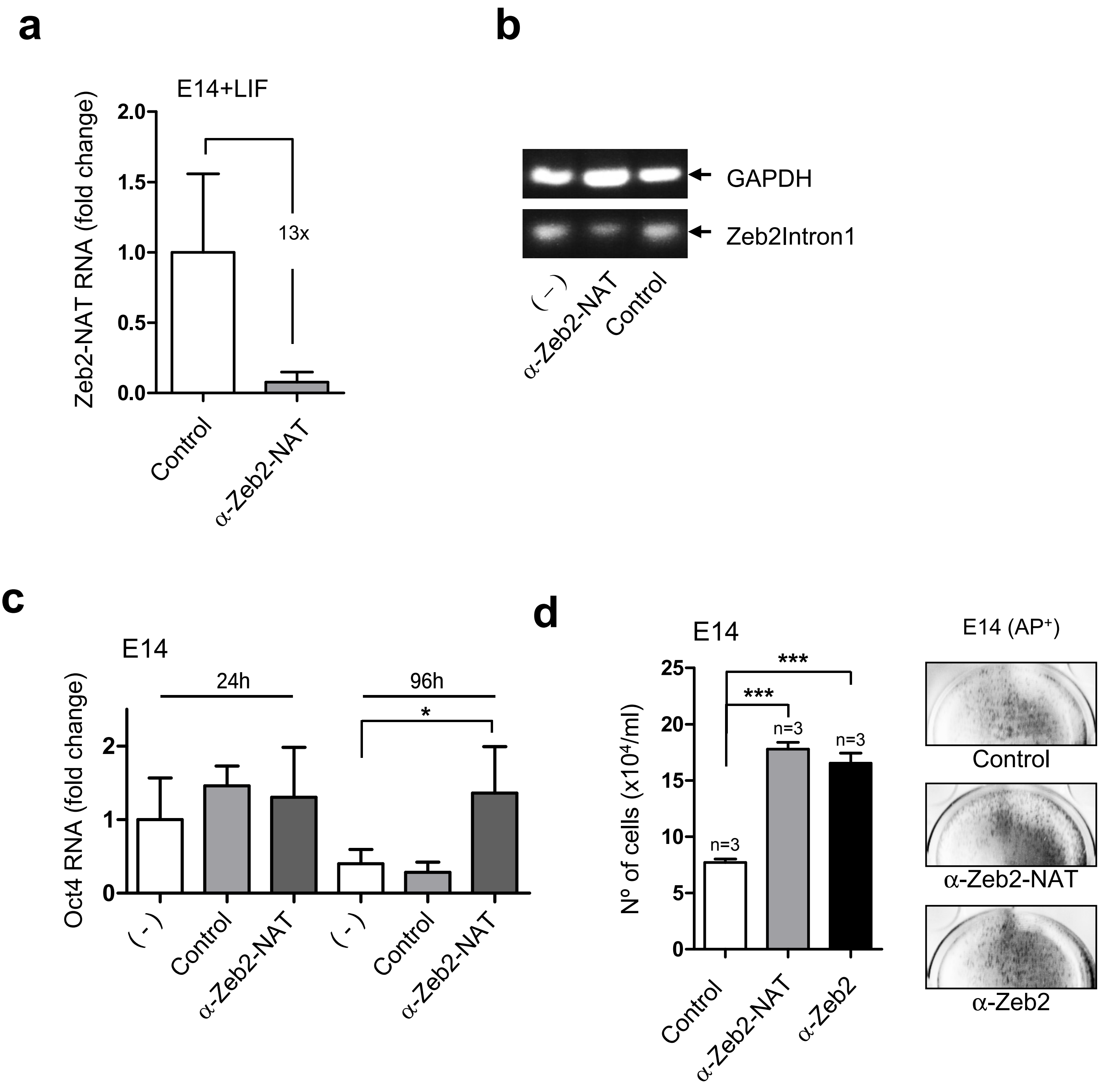

Supplementary Figure 10. Downregulation of Zeb2-NAT expression in stem cells. a, qRT-PCR analysis of Zeb2-NAT transcripts in E14 cells that were transfected with either control LNA gapmers or oligonucleotides targeting Zeb2-NAT. **b**, Semi-quantitative RT-PCR analysis of GAPDH and Zeb2 transcripts with retention of the first intron in E14 cells that were either mock transfected (-) or transfected with anti-Zeb2-NAT and control LNA gapmers. **c**, qRT-PCR analysis of Sox2 and Oct4 transcripts in E14 cells that were transfected as indicated and maintained for 24h and 96h in feeder-free conditions without 2i. Transcript levels were normalized to GAPDH mRNA and depicted as fold change. **d**, Quantification of AP^+ clones: the same number of E14 cells transfected with either control LNA gapmers or oligonucleotides targeting Zeb2-NAT or Zeb2 were maintained for 4 days in feeder-free conditions without 2i; cells were then stained for alkaline phosphatase (AP) activity.

Fig 1g

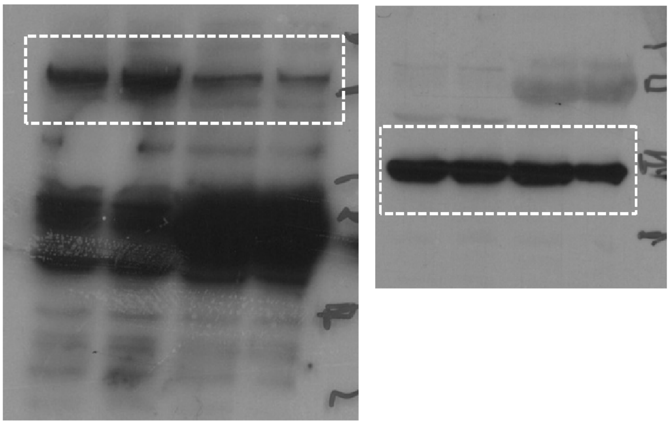

Fig 3c

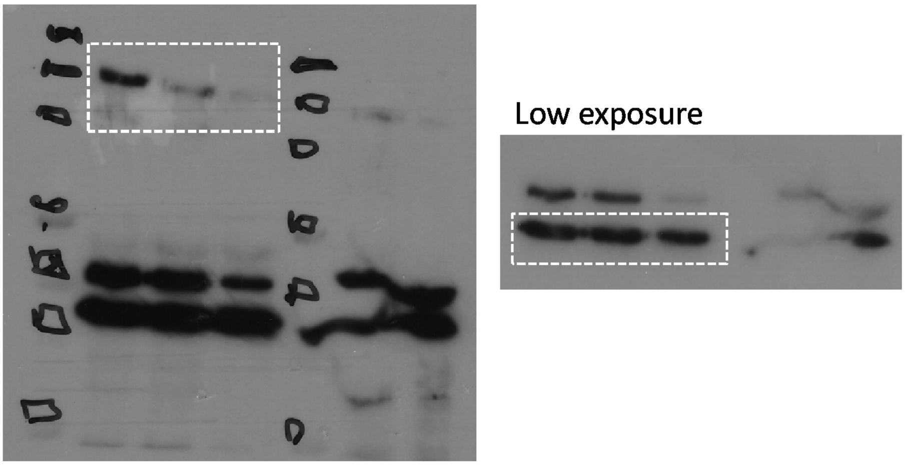

Fig 5d

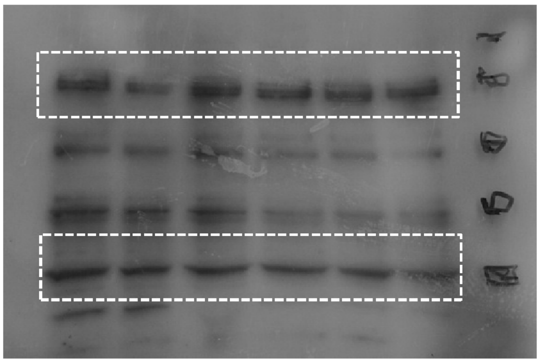

Fig 6b

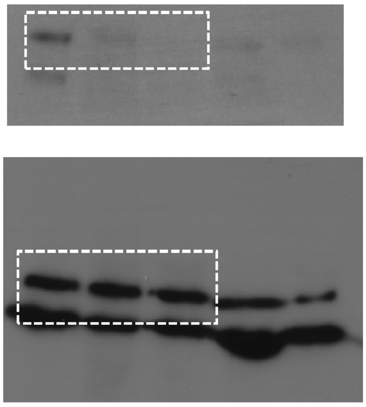

Fig 7d & S8c

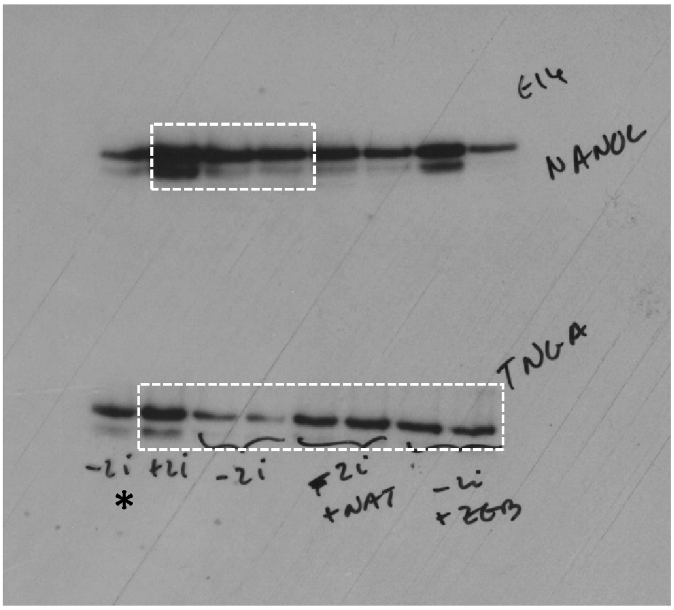

* - 24h -2i condition

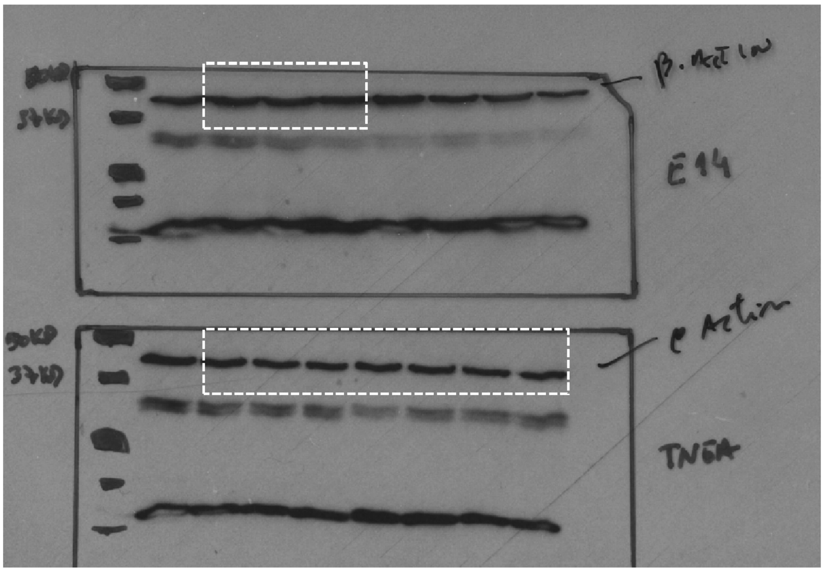

Fig S5c

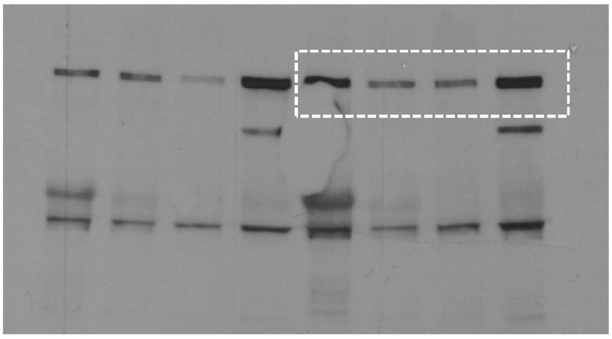

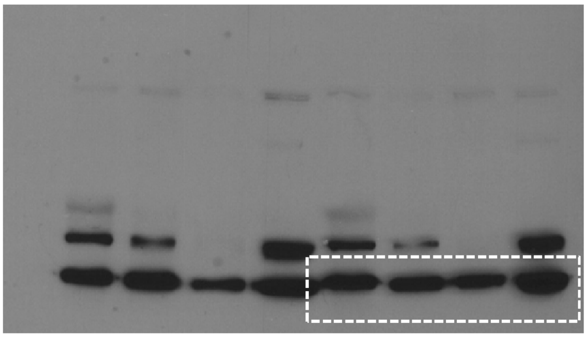

Supplementary Figure 11. Uncropped western blots.

Supplementary Table 1. Sequences.

LNA Gapmers

Target RNA	Sequence
Zeb2	**5' – CAC**GTTAGCCTGAGAGGAG**GTT-3' ***
Zeb2-NAT	**5' – CAC**ACTCTGCAGGATTTA**GTT-3' ***
Zeb2-NAT	**5' – CAC**TTAGTGATGAGGATA**GTT-3' ***

* The center (gap) of the antisense oligonucleotides consist of deoxynucleotide bases and phosphorothioate backbone linkages. The flanking LNA-modified bases are depicted in bold.

2'OMe RNAs

Target RNA	Sequence
mZeb2-NAT #1	**5' – [mG][mC][mU][mU][mU][mG][mC][mG][mG][mA][mA][mA] [mA][mC][mC][mU][mG][mG][mA][mA][mA] -3'**
mZeb2-NAT #2	**5' – [mA][mA][mA][mG][mG][mU][mG][mG][mA][mG][mG][mC] [mG][mA][mA][mG][mA][mA][mA][mC]-3'**

shRNAs

Target RNA	Sequence	Particle titer
mZeb2	**CCCATTTAGTGCCAAGCCTTT**	**8.7×10^6 TU/ml**
mZeb2	**CCACTAGACTTCAATGACTAT**	**1.3×10^7 TU/ml**
mZeb2-NAT	**ATGCAGATCTCTTGTCTTATA**	**9.4×10^6 TU/ml**
mZeb2-NAT	**AGGGATTGGTTATGCAAATAT**	**1.2×10^7 TU/ml**
	**Non-Target Control**	**7.9×10^6 TU/ml**

Supplementary Table 2. Primers used for qRT-PCR

Target RNA	Forward primer	Reverse primer	Product Size (bp)
Actin	GGCACCACACCTTCTACAATG	GTGGTGGTGAAGCTGTAG	352
GAPDH	TTCACCACCATGGAGAAGGC	CCCTTTTGGCTCCACCCT	52
Zeb2 #1	TATGGCCTATACCTACCCAAC	AGGCCTGACATGTAGTCTTGTG	126
Zeb2-NAT #1	ACAAAGATAGGTGGCGCGTG	GCATGAAGAAGCCGCGAAGTGT	271
Zeb2-NAT #2	CTGGACCCCTCTACACCTCA	CCAATCCCTTCAGAGCAAAG	213
Zeb2-intron	CGTGTGCATTCCCTCATACG	CTGTTTGGTGTGTTGCACTC	94
mOCT4	TAGGTGAGCCGTCTTTCCACC	GCTTAGCCAGGTTCGAGGAT	160
mSOX2	GCGGAGTGGAAACTTTTGTCC	CGGGAAGCGTGTACTTATCCTT	157
mNANOG	AGGGTCTGCTACTGAGATGCTCTG	CAACCACTGGTTTTTCTGCCACCG	363
mECad	AATGGCGGCAATGCAATCCCAAGA	TGCCACAGACCGATTGTGGAGATA	93
mp16	CGTACCCCGATTCAGGTGAT	TTGAGCAGAAGAGCTGCTACGT	59
mp21	GGCCCGGAACATCTCAGG	AAATCTGTCAGGCTGGTCTGC	52
mp53	CCCCTGTCATCTTTTGTCCCTT	GGGAGGAGAGTACGTGCACATAA	114
mZeb2 A	CCACATTGTCGCTGTGTTTG	CCCGGCTCACTTCAGACTA	150
mZeb2 B	GCCATCTGATCCGCTCTTAT	GGCTTCCTTCTCCCTGTCC	179
mZeb2 C	TATGTGGGGGCATTGGTAT	GAGGGTTTGCAAGGCTAT	157
mZeb2 D	CGACACGGCCATTATTTACC	ATGAAATTCCATGCCTCTGC	208
mZeb2 E	ACCTTTTTCTCCCCCACACT	CGGCTGCTTCATTGATAAGA	166
mZeb2 F	GCCATCTGATCCGCTCTTAT	GAGGGTTTGCAAGGCTATCA	188

Supplementary Table 3. Antibodies used for immunoblotting and immunofluorescence

Target protein	Supplier	Dilution Immuno-blotting	Dilution Immuno-fluorescence
SSEA-1	MAB4301 - Millipore		1:250
Nanog	Alexa fluor 488 conjugated - eBioscience	1:500	1:200
Zeb2	ABE573 - Millipore	1:500	1:200
beta-Catenin	180226 - clone CAT-5H10 - ThermoFisher		1:150
